# Antibiotic treatment of acute uncomplicated cystitis based on rapid urine test and local epidemiology: lessons from a primary care series

**DOI:** 10.1186/1471-2334-14-137

**Published:** 2014-03-11

**Authors:** Manuel Etienne, Emmanuel Lefebvre, Noëlle Frebourg, Hélène Hamel, Martine Pestel-Caron, François Caron

**Affiliations:** 1 Infectious diseases, Rouen University Hospital, rue de Germont, Rouen F-76031, France; 2 Research Group on Antimicrobials (GRAM-EA2656) IRIB, Rouen University Hospital, 22 boulevard Gambetta, Rouen 76000, France; 3 Primary care department, Medical Faculty, 22 boulevard Gambetta, Rouen 76000, France; 4 Bacteriology, Rouen University Hospital, rue de Germont, Rouen F-76031, France; 5 Clinical research center, Rouen University Hospital, rue de Germont, Rouen F-76031, France

**Keywords:** Acute uncomplicated cystitis, Urinary tract infection, Primary care, Rapid urine test, Antibiotic policy

## Abstract

**Background:**

Acute uncomplicated cystitis (AUC) is an ideal target of optimization for antibiotic therapy in primary care. Because surveillance networks on urinary tract infections (UTI) mix complicated and uncomplicated UTI, reliable epidemiological data on AUC lack. Whether the antibiotic choice should be guided by a rapid urine test (RUT) for leukocytes and nitrites has not been extensively studied in daily practice. The aim of this primary care study was to investigate local epidemiology and RUT-daily use to determine the optimal strategy.

**Methods:**

General practitioners included 18–65 years women with symptoms of AUC, performed a RUT and sent urines for analysis at a central laboratory. Different treatment strategies were simulated based on RUT and resistance results.

**Results:**

Among 347 enrolled patients, 78% had a positive urine culture. *Escherichia coli* predominated (71%) with high rates of susceptibility to nitrofurantoin (100%), fosfomycin (99%), ofloxacin (97%), and even pivmecillinam (87%) and trimethoprim-sulfamethoxazole (87%). Modelization showed that the systematic use of RUT would reduce by 10% the number of patients treated. Fosfomycin for patients with positive RUT offered a 90% overall bacterial coverage, compared to 98% for nitrofurantoin. 95% for ofloxacin, 86% for trimethoprim-sulfamethoxazole and 78% for pivmecillinam.

**Conclusion:**

Local epidemiology surveillance data not biased by complicated UTI demonstrates that the worldwide increase in antibiotic resistance has not affected AUC yet. Fosfomycin first line in all patients with positive RUT seems the best treatment strategy for AUC, combining good bacterial coverage with expected low toxicity and limited effect on fecal flora.

**Trial registration:**

The current study was registered at clinicaltrials.gov (NCT00958295)

## Background

The emergence of multidrug resistant *Enterobacteriaceae* in the community reinforces the need to reduce antibiotic prescription, particularly for molecules at high risk of ecological effect such as broad spectrum beta-lactams and quinolones. Given its benign nature, acute uncomplicated cystitis (AUC) is an ideal target of action. Rapid urine test (RUT) for detection of nitrites and leucocytes have a high negative predictive value, so that some guidelines on AUC recommend their systematic use, such as the French [1], the Scottish [2] and that of the European Association of Urology [3]. Of note, diagnostic procedures are not addressed by the current IDSA and ESCMID guidelines [1,4]. To spare beta-lactams and quinolones, it is now recommended worldwide to treat AUC with nitrofurantoin, trimethoprim-sulfamethoxazole (TMP-SMX), fosfomycin or pivmecillinam, depending on local community resistance prevalence [1,4]. Unfortunately, as underlined by the IDSA and ESCMID guidelines, local epidemiological data on AUC lack, and surveillance networks mix complicated and uncomplicated urinary tract infection (UTI) [5]. In France, fosfomycin is the only drug recommended first line for AUC; because of its poor efficacy against *Staphylococcus saprophyticus*, it is suggested, but with a low level of evidence, that patients under 30 years (at higher risk of *S. saprophyticus* infection) and with negative nitrite detection should be treated with nitrofurantoin or quinolones.

Thus, the current study was performed to evaluate the current epidemiology of AUC, the contribution of RUT in a primary care practice, and to compare different treatment strategies.

## Methods

### Patients and ethical issues

Patients were enrolled from 2009 to 2011, a network of French general practitioners (GPs). Patients included were those routinely diagnosed AUC in primary care, and had the following inclusion criteria: female gender, age between 18 and 65 years, and pain on urination, pollakiuria, urgency, or supra-pubic pain. Patients with complicated or recurrent cystitis or pyelonephritis were excluded. Thus, urinary tract disorders (such as renal failure, lithiasis, reflux, urinary catheter etc.), diabetes, immune deficit, cancer, recurrent urinary tract infections (≥3 episodes in the previous year), and pregnancy were causes of exclusion. Patients with fever or flank pain were also excluded. All the patients meeting the inclusion criteria gave informed consent. For each patient included, the GP received an honorarium equivalent to the cost of a consultation (21 Euros) to compensate for the time needed for the study. The study was approved by the local protection committee (CPP-SC 2009/003), and registered at clinicaltrials.gov (NCT00958295) and funded by Rouen University Hospital.

### Urine analysis

At the GP’s office, a midstream urine sample was collected after perineal cleansing, using the BD Vacutainer® urine collection and transport medium kit. RUT, to detect leukocytes and nitrites (Bayer multistix®), was immediately performed by the GP.

The urine sample was then analyzed within 48 hours at the Rouen University Hospital laboratory according to French recommendations [6-10]. Significant threshold was >10^4^/mL for leukocyte count. Two threshold were used for bacterial count after urine culture: (a) “standard thresholds”, i.e., those of the current French guidelines ≥10^3^ CFU/mL for *Enterobacteriacae* and *S. saprophyticus*, ≥10^5^ CFU/mL for other pathogens [1,11]; (b) and “reduced thresholds”, used in other studies on AUC: ≥10^2^ CFU/mL for *Enterobacteriacae* and *S. saprophyticus*, ≥10^4^ CFU/mL for other pathogens [4,6,8-22]. Urine cultures growing more than two bacteria were considered contaminated. Pathogens were identified based on their biochemical reaction according to the API system (BioMerieux®, La Balme les grottes, France). Susceptibility testing was performed by disk diffusion method and strains were classified as susceptible, intermediate, or resistant according to French recommendations [1,4,12,23-25].

Results of urine analysis were sent to GP only on request since urine analysis is recommended in France only in case of failure of the probabilistic treatment.

### Antibiotic strategies

The GP decided whether to prescribe antibiotics or not, based on its own judgment (no recommendations in the study protocol).

To analyze the contribution of RUT to the antibiotic decision, different antibiotic strategies were modelized for the total cohort, based on RUT and culture results. The first strategy evaluated administration of fosfomycin, nitrofurantoin, TMP-SMX, pivmecillinam or ofloxacin to all patients (i.e., no RUT used). The second strategy evaluated different antibiotic treatments solely in patients with leukocyte or nitrite positive RUT. The antibiotic choice could be fosfomycin, TMP-SMX, or pivmecillinam in all cases; alternatively, fosfomycin could be indicated for all cases, excepted women <30 years with negative nitrite detection by RUT for which nitrofurantoin or ofloxacin were preferred.

For each treatment strategy, three parameters were estimated: (1) the rate of patients treated with antibiotics; (2) the rate of appropriate decisions to prescribe antibiotics or not: the decision was qualified appropriate when the GP prescribed antibiotics to a patient with positive urine culture or did not prescribe antibiotics to a patient with negative culture; conversely, the decision was qualified inappropriate when the GP prescribed antibiotics to a patient with negative culture or did not prescribe antibiotics to a patient with positive culture; (3) the overall rate of bacterial coverage, measured only in patients with AUC confirmed by urine culture, and depending on distribution of pathogens and their resistance rates.

### Statistical methods

The results were analyzed *per protocol*, described by their mean value and standard deviation, or by frequency. Quantitative data were compared using the Fisher exact *t* test. A *p* value <0.05 was considered significant.

## Results

### Population

A total of 362 patients were included, but 15 patients were excluded for recurrent cystitis (n = 3), delays in transportation to the laboratory (n = 11), or both (1). Mean age was 38 years with two peaks of incidence, women aged 18 to 29 years and 42 to 53 years accounting for 32% and 38% respectively of the cohort.

### Rapid urine test for detection of leukocytes and nitrites

RUT was performed in all 347 cases, detecting leukocytes (L) in 302 (87%), and nitrites (N) in 81 cases (23%), with the following results: L + N + = 74 (21%), L-N- = 37 (11%), L-N + = 7 (2%), L + N- = 229 (66%).

### Urine analysis

Table 1 shows the results and leukocyte counts and bacterial cultures. For 27 patients (8%), no significant leukocytes and no significant bacteriuria were observed. Significant leukocyte concentration in urine was observed in 305 patients (88%). With standard thresholds, 199 (57%) cultures were positive for 1 (n = 194) or 2 pathogens (n = 5). With reduced thresholds, 272 (78%) cultures were positive for 1 (n = 266) or 2 (n = 6) pathogens. Only 33 urine cultures (9.5%) had no visible growth. Among the pathogens isolated *Escherichia coli* dominated (n = 157, 77%) while *S. saprophyticus* was at the second rank (n = 14, 7%) followed by *Proteus* spp (n = 10, 5%), *Enterococcus spp* (n = 9, 4.4%), *Klebsiella spp* (n = 5, 2.5%), *Enterobacter spp* (n = 5, 2.5%), and *Citrobacter spp* (1), *Streptococcus spp* (1) and *S. aureus* (1). Among the 14 patients with *S. saprophyticus* infection, eight (57%) were less than 30 years old. No significant change was observed in overall distribution of the species analyzed when reduced thresholds were considered.

**Table 1:** Results of the rapid urine test (RUT) performed by the general practitioner compared to urine analysis at the central laboratory

**RUT results**	**Urine analysis**		
	**Leukocyturia**^**a**^	**Bacteriuria**	
		**at standard thresholds**^**b**^	**at reduced thresholds**^**c**^
L+ / N + (n = 74)	73 (99%)	61 (82%)	70 (95%)
		58 *Enterobacteriaceae*	62 *Enterobacteriaceae*
		3 *S. saprophyticus*	4 *S. saprophyticus*
			5 miscellaneous
L- / N- (n = 37)	9 (24%)	3 (8%)	6 (16%)
		3 *Enterobacteriaceae*	6 *Enterobacteriaceae*
			2 miscellaneous
L+ / N- (n = 229)	216 (94%)	128 (56%)	189 (83%)
		114 *Enterobacteriaceae*	162 *Enterobacteriaceae*
		9 *S. saprophyticus*	11 *S. saprophyticus*
		9 miscellaneous	18 miscellaneous
L- / N + (n = 7)	7 (100%)	7 (100%)	7 (100%)
		6 *Enterobacteriaceae*	6 *Enterobacteriaceae*
		2 *S. saprophyticus*	2 *S. saprophyticus*
		2 miscellaneous	
Total (n = 347)	305 (88%)	199 (57%)	272 (78%)
		178 *Enterobacteriaceae* (89%)	236 *Enterobacteriaceae* (85%)
		14 *S. saprophyticus* (7%)	17 *S. saprophyticus* (6%)
		11 miscellaneous (5%)	25 miscellaneous (9%)

^a^Leukocyturia >10^4^/mL considered significant.

^b^Standard thresholds: ≥10^3^ CFU/mL for *Enterobacteriaceae* and *S. saprophyticus*, ≥10^5^ CFU/mL for other pathogens.

^c^Reduced thresholds: ≥10^2^ CFU/mL for *Enterobacteriaceae* and *S. saprophyticus*, ≥10^4^ CFU/mL for other pathogens.

Table 2 describes the epidemiology of resistance for *E. coli*, all Gram negative, all Gram positive, and for all pathogens together. Globally, an extremely low rate of resistance was observed. In particular, no extended-spectrum beta-lactamase (ESBL) producing enterobacteria and no multi-drug resistant organisms were isolated. Among *E. coli* isolates, only one strain expressed a cephalosporinase, and a very low rate of quinolone resistance was observed (ofloxacin resistance: 2%), even at a low-level (nalidixic acid resistance: 3%). The most common *E. coli* resistance phenotypes were wild type (56%), amoxicillin resistance (27%), or amoxicillin + TMP-SMX resistance (12%). Overall susceptibility rates of the isolated pathogens were above 85% for the antibiotic regimen most commonly used to treat AUC: fosfomycin, nitrofurantoin, TMP-SMX, or fluoroquinolones, and 75% for pivmecillinam.

**Table 2:** Antibiotic susceptibility of the pathogens isolated at standard thresholds from urine culture of acute uncomplicated cystitis

**Antibiotic**	**Antibiotic susceptibility**^**a **^**(%)**			
	***E. coli***	**All Gram -**	**All Gram +**	**All pathogens**
	**(n = 157)**	**(n = 178)**	**(n = 25)**	**(n = 203)**
Amoxicillin	63	56	32	/
Amox/clav	91	88	NT^b^	/
Ticarcillin	64	61	NT	/
Ticar/clav	92	92	NT	/
Piperacillin	64	61	NT	/
Pip/Taz	99	99	NT	/
Pivmecillinam	87	88	NT	76^c^
Cefalotin	63	61	NT	/
Cefotaxime	99	99	NT	/
Ceftazidime	99	99	NT	/
Cefepime	99	99	NT	/
Imipenem/cilastatin	100	100	NT	/
Gentamicin	99	99	57^d^	/
Tobramycin	99	99	NT	/
Amikacin	100	100	NT	/
Netilmicin	99	99	NT	/
TMP-SMX	87	87	63	85
Nalidix acid	97	87	NT	/
Ofloxacin	97	99	59	94
Levofloxacin	97	99	95	98
Nitrofurantoin	100	92^e^	100	98
Fosfomycin	99	98	27	89
Fucidic acid	NT	NT	58	/
Erythromycin	NT	NT	17	/
Lincomycin	NT	NT	62	/
Pristinamycin	NT	NT	62	/
Rifampin	NT	NT	61	/
Vancomycin	NT	NT	100	/

^a^Intermediate strains considered resistant.

^b^NT: not tested.

^c^All Gram + were considered resistant *in vitro* to pivmecillinam.

^d^Natural low level resistance of *Streptococcus* and *Enterococcus spp* considered non susceptible.

^e^*Proteus spp* considered naturally resistant to nitrofurantoin.

### Antibiotic treatments

During the previous 6 months, 91 patients (26%) had received antibiotics for any reason, either at one (n = 72, 79%), two (n = 14, 15%), or three occurrences (n = 5, 5%) during an average of 6.7 days. No obvious correlation between previous exposure to antibiotics and resistance to urine pathogen was observed. Of the 347 patients, 31 (9%) were not treated with antibiotics. RUT was negative for both leukocyte and nitrite detection for 25 of them, negative for leukocytes in four patients, and negative for nitrites in two patients. Of the 316 patients treated with antibiotics, 148 (47%) had a bacteriuria below the standard thresholds, and 94 patients (30%) below reduced thresholds. While French guidelines recommend fosfomycin (single dose) as first-line, and nitrofurantoin (5 days) or fluoroquinolones (≤3 days) only as second-line treatment, GPs chose almost equally fosfomycin (n = 150, 47%) and fluoroquinolones (n = 142, 45%), and rarely cefixim (n = 10, 3%), nitrofurantoin (n = 8, 2.5%) or TMP-SMX (n = 6.2%).

### Diagnosis performance of RUT versus urine culture

Table 3 shows the performance of RUT compared to urine culture with two different bacterial thresholds in this population of female patients with symptoms suggestive of AUC. RUT had a negative predictive value of 92% when standard thresholds were used, lessening to 84% with reduced thresholds. Hypothetically, had GPs decided to treat only patients with leukocytes or nitrites detected at RUT, 310 patients (89%) would have been treated. Of these 310 patients, 115 patients (37%) would not finally be diagnosed with AUC according to urine culture at standard thresholds and 60 (19%) at reduced thresholds. Of the 37 non treated patients (11%), 6 patients (16%) would finally be diagnosed with cystitis according to urine culture at standard thresholds and 7 (19%) at reduced thresholds.

**Table 3:** Performance of the rapid urine test for the diagnosis of AUC according to the bacterial concentration in urine culture

**Performance criteria**	**Bacterial concentrations in urine cultures**	
	**≥ standard thresholds**^**a**^	**≥ reduced thresholds**^**b**^
Sensitivity	98%	98%
Specificity	23%	33%
Positive predictive value	63%	79%
Negative predictive value	92%	84%

^a^Standard thresholds: ≥10^3^ CFU/mL for *Enterobacteriaceae* and *S. saprophyticus*, ≥10^4^ CFU/mL for other pathogens.

^b^Reduced thresholds: ≥10^2^ CFU/mL for *Enterobacteriaceae* and *S. saprophyticus*, ≥10^3^ CFU/mL for other pathogens.

Of the 266 patients with leukocyte positive and nitrite negative RUT, *Enterobacteriaceae* were isolated in 117 cases (44%), mostly with a concentration ≤10^3^ CFU/mL and *S. saprophyticus* was isolated in only 9 cases (3%). In the subgroup of patients below 30 years of age (n = 90), *S. saprophyticus* was isolated in only six of them (15%). For detection of *Enterobacteriaceae*, nitrite detection by RUT had 11% sensitivity, 93% specificity, 74% positive predictive value, and 35% negative predictive value. RUT performance in detecting *Enterobacteriaceae* was not significantly affected by reduced diagnosis thresholds for diagnosis in urine culture (data not shown).

### Modelization of treatments

Table 4 details the results of the modelized treatment strategies. Use of RUT to help GPs in decision-making regarding antibiotic prescription lowered the number of patients treated by 10%, and significantly improved the appropriateness of antibiotic prescription (73% *vs* 80%, *p* = 0.02), regardless of bacterial thresholds used. As expected, given the low ability of RUT to predict *S. saprophyticus* infection, nitrite-detection-based strategies did not improve overall success rates. Whichever antibiotic strategy was simulated, overall bacterial coverage rates exceeded 85% in all cases, except in pivmecillinam-based strategies (≥76%). Even TMP-SMX-based strategies had ≥85% presumed bacterial coverage. Nitrofurantoin or fluoroquinolone-based strategies had the highest rates of bacterial coverage, achieving 4 to 6% improvement on fosfomycin-based strategies.

**Table 4:** Evaluation of different treatment strategies based on local epidemiology and on the results of rapid urine test (RUT) in a cohort of 346 patients with symptoms of acute uncomplicated cystitis

	**Antibiotic (ATB) Treatment strategy**		
	**ATB for all symptomatic patients (*i.e. *** **no RUT used)**	**ATB only in patients with positive RUT**^**a**^	
Patients treated (%)	347 (100)	311 (89)	
**Appropriate ATB decision**^b^**(%)**	253 (73)	278 (80)	0.02^**d**^
No ATB, negative UC^c^	0	31	
ATB, positive UC	253	247	
**Inappropriate ATB decision (%)**	94 (27%)	70 (20%)	
ATB, negative UC	94	64	
No ATB, positive UC	0	6	
**Overall susceptibility rates according to the ATB regimen**^**e**^**(%)**			
Fosfomycin	89	90	
Nitrofurantoin	98	98	
TMP-SMX	85	86	
Ofloxacin	94	95	
Pivmecillinam	76	78	
Fosfomycin, except if RUT-			
For nitrites and age < 30: nitrofurantoin		93	
Fosfomycin, except if RUT-			
For nitrites and age < 30: ofloxacin		93	

^a^RUT considered positive when leukocytes and/or nitrites were detected.

^b^ATB decision considered appropriate or not according to the results of urine analysis.

^c^UC: urine culture.

^d^NB: p = 0.02 when appropriate and inappropriate ATB decision were compared using standard thresholds, and p = 0.03 using reduced thresholds.

^e^Susceptibility rate for patients with a positive urine culture.

## Discussion

Three main messages can be learnt from this series performed at the GP’s office in a strict “real-life” clinical setting: (a) the rate of antibiotic resistance remains extremely low in this specific population of female patients with non recurrent AUC, so that fluoroquinolones, fosfomycin, nitrofurantoin, TMP-SMX, and to a lesser extent pivmecillinam, offer excellent bacterial coverage; (b) the bacterial thresholds retained in the guidelines for biological diagnosis derive from specific studies mainly on inpatients, and might not fit daily care reality; (c) rapid urine test can reduce inappropriate antibiotic prescriptions by 10%, but cannot accurately suspect *S. saprophyticus* infections. From these results, we suggest that RUT and recent epidemiology data offer prescribers the rare opportunity both to optimize and to reduce antibiotic prescription, hence lowering risk of adverse ecological effects.

Optimal probabilistic antibiotic prescription requires recent and detailed epidemiological data. In this series, very few levels of resistance were observed (only 3% of resistance to fluoroquinolone and 1% of resistance to third generation cephalosporin for *E. coli*). Resistance rates in our series are very similar to recent international studies specifically devoted to AUC [1,4,26-31]. This can probably be explained by the fact that women with non recurrent AUC are otherwise healthy subjects, uncommonly exposed to antibiotics and thus to any ecological adverse effect on their gut flora. Indeed only a quarter of our patients had received antibiotics during the previous six months, a rate twice lower than that of the general population of 18–65 years old [32]. Our results markedly differ from those of French and international surveillance networks that reported during the same period resistance for outpatient urinary *E. coli* isolates up to 14% for fluoroquinolones and up to 5% for third generation cephalosporin. These latter networks are biased, because they include patient cohorts visiting urologists, or patients with recurrent or complicated UTI that have higher antibiotic pretreatments hence are at higher risk of multidrug resistance [1,6-10,33-35]. Hence global epidemiology data UTI should not be used to guide antibiotic choice for AUC.

The second prerequisite for improving the accuracy of antibiotic prescription is using reliable diagnosis methods. As for AUC diagnosis can rely on symptoms, RUT, or urine analysis. Numerous studies have proposed scores based on clinical symptoms and RUT, all of them using urine analysis as the gold standard. In these studies the pretest likelihood of infection increased RUT performance [4,12-22]. In our study, since all patients were young females without comorbidities, consulting for symptoms suggesting AUC, it was assumed that the probability of AUC exceeded 80% when RUT detected nitrites or leukocytes [1,4,12]. Using the standard bacterial thresholds retained in French guidelines (≥10^3^ CFU/mL for *Enterobacteriaceae* or *S. saprophyticus*, and ≥10^5^ CFU/mL for other pathogens), 148 (43%) patients did not have the biological criteria for diagnosis of AUC [1,4]. A high rate of AUC differential diagnosis (e.g., vaginitis or urethritis) is most unlikely, and we suspect that standard thresholds do not fit reality. Indeed, standard thresholds have been determined from morning concentrated urines [6-10], whereas in our study as in daily life, urine was mostly collected during daytime so that significant thresholds were certainly lower. This explanation is supported by the high rate of significant leukocyturia (82% of our patients) while no visible growth was uncommon (only 9.5% of patients). Using reduced thresholds for all pathogens, 247 urine analyses (71%) would have been positive, which is more consistent with other studies. This debate on diagnosis thresholds is crucial for evaluating RUT performances for diagnosis of AUC. As a fact, depending on the diagnosis thresholds used, the negative predictive value of RUT lowered from 92% to 84%. But, more meaningful for the prescriber than the negative predictive value of RUT, may be evaluation of the impact of RUT-based strategies on the appropriateness of decision-making regarding antibiotic prescription: if GPs had prescribed antibiotics only in patients with leukocyte or nitrite positive RUT, antibiotic prescription would have been reduced by 10% and the appropriateness of the decision improved, regardless of diagnosis thresholds used. Of those patients with biologically confirmed AUC, only three to six out of 347 patients would not have been treated with antibiotics, but, according to data from the literature, up to three patients might have been cured spontaneously, or all six patients could have consulted later with minimal risk of progression to pyelonephritis [1,11]. RUT however cannot help in identifying the causative pathogen. Contrary to French guideline suggestions, RUT demonstrated low ability to discriminate between *Enterobacteriaceae* and Gram positive pathogens, since patients were more likely to have a Gram positive infection when nitrite detection was positive than negative. Even taking into account the subgroup of patients below 30 years old, in which prevalence of *S. saprophyticus* is higher, Gram positive only represented 15% of patients. Thus, we recommend that the diagnosis thresholds retained in urine analysis be tempered, and that RUT be used to limit inappropriate antibiotic prescriptions, not to anticipate microbiological diagnosis.

As a consequence of the low resistance rates, bacterial coverage rate was high for all antibiotics evaluated, with only a slight superiority for nitrofurantoin or fluoroquinolones. Bacterial coverage rate can only be cautiously considered as an indirect, and probably underestimated evaluation of the cure rate of antibiotic treatment for AUC, because of spontaneous cure in up to 40% of untreated patients, and because of high antibiotic urine concentrations that may lead to cure despite *in vitro* resistance [6,8-11]. However, bacterial coverage rate might be accurate for comparing antibiotic regimen such as nitrofurantoin and fosfomycin which demonstrated similar clinical and microbiological efficacy on susceptible strains in previous studies [23-25]. In our study, both molecules exhibited high (≥95%) and similar rates of bacterial coverage. Pivmecillinam had the lowest rate of bacterial coverage, estimated by *in vitro* susceptibility data, because Gram positive are considered naturally resistant. Nevertheless, several clinical studies have demonstrated up to 93% clinical cure rate in *S. saprophyticus* AUC because of high pivmecillnam concentration in urine [26-31]. If pivmecillinam had been considered active *in vivo* on *S. saprophyticus* infections, the estimated success rates of pivmecillinam treatments would have reached 81% in our series.

Since all simulated antibiotic strategies appeared highly effective, priority should be given to the risk of adverse effects, including ecological damage. Among the for antibiotics currently recommended the 2010 IDSA and ESCMID guidelines, we consider that fosfomycin has the best benefit-risk balance, at the individual and collective levels; pivmecillinam though well tolerated requires a heavier regimen (bid for 3–5 days) for potentially a lower efficacy; TMP-SMX causes early and sustained ecological damage as well as rare but serious toxicity [36]; nitrofurantoïn is proposed first-line by many countries, because of good coverage rate and low ecological pressure. Because severe adverse effects such as allergic pneumonia or hepatitis and neuropathy have rarely been described even after a very short course, we suggest it should be used second-line [1,33].

## Conclusions

In conclusion despite worldwide increase in the prevalence of resistance for complicated UTI, patients with AUC still exhibit extremely low resistance rates. Systematic use of rapid urine test could reduce, the amount of antibiotics prescribed by 10%. Whenever required, antibiotics should be chosen exclusively from molecules that have minimal adverse effects including collateral damages.

## Abbreviations

AUC: Acute uncomplicated cystitis; UTI: Urinary tract infection; RUT: Rapid urine test; IDSA: Infectious diseases society of America; ESCMID: European Society for Clinical Microbiology and Infectious Diseases; TMP-SMX: Thrimetoprime-sulfamethoxazole; GP: General practitionner; L: Leukocytes; N: Nitrites; ESBL: Extended spectrum beta-lactamase.

## Competing interests

The authors declared that they have no competing interests.

## Authors’ contributions

ME designed and implemented the study, analyzed the results and wrote the manuscript. EL coordinated the network of general practitioners, included patients, and revised the manuscript. NF analyzed all the urine samples at the central laboratory, and revised the manuscript. HH collected, supervised and checked all the data, and managed all the logistics. MPC contributed in the design of the study, participated in the analysis of urine samples, and revised the manuscript. FC supervised the whole study, and substantially revised the manuscript. The Bacyst study group includes all the general practitioners that included patients in the study, collected data, and sent the urine samples to the central laboratory. All authors read and approved the final manuscript.

## Authors’ information

Bacyst study group: Philippe André, Alain Barbot, Nicole Bénard, Danièle Bouillon, Pascal Boulet, Thomas Bourez, Laurence Brenet, Jean-Michel Bunel, Martine Courtier, François De Golmard, Marc Durand, Pierre Fainsilber, Rodolphe Hautot, Jean-Loup Hermil, Pascal Julienne, Marie-Catherine Lagaude, Karine Larese, Laurent Laval, Rrené Le, Janick, Lefebvre, Laure Lefebvre, Serge Lejeal, Jérome Longueville, Jean-Pierre Mineo, Philippe Nguyen-than, Xavier Odoux, Marc-Henri Othman, Roseline Peluchon, Marc Salaun, Sébastien Taupin, Jean-Paul Thueux, Françoise Valla, Jean Van Elslande.

## Pre-publication history

The pre-publication history for this paper can be accessed here:

http://www.biomedcentral.com/1471-2334/14/137/prepub

## References

[B1] AFSSAPS (Agence française de sécurité sanitaire des produits de santé) AFSSAPS Practice recommendations for diagnosis and antibiotic therapy of adult community urinary tract infections Med Mal Infect 2008 14 S203 S252 19209393

[B2] Scottish Intercollegiate Guidelines Network Management of suspected bacterial urinary tract infection in adults: a national clinical guideline 2012 1 52 Available at: http://www.sign.ac.uk/pdf/sign88.pdf

[B3] Naber KG Bergman B Bishop MC Bjerklund-Johansen TE Botto H Lobel B Jinenez Cruz F Selvaggi FP Urinary Tract Infection (UTI) Working Group of the Health Care Office (HCO) of the European Association of Urology (EAU) EAU guidelines for the management of urinary and male genital tract infections. Urinary Tract Infection (UTI) Working Group of the Health Care Office (HCO) of the European Association of Urology (EAU) Eur Urol 2001 14 5 576 588 10.1159/000049840 11752870

[B4] Gupta K Hooton TM Naber KG Wullt B Colgan R Miller LG Moran GJ Nicolle LE Raz R Schaeffer AJ Soper DE Infectious Diseases Society of America, European Society for Microbiology and Infectious Diseases: International clinical practice guidelines for the treatment of acute uncomplicated cystitis and pyelonephritis in women: A 2010 update by the Infectious Diseases Society of America and the European Society for Microbiology and Infectious Diseases Clin Infect Dis 2011 14 e103 e120 21292654 10.1093/cid/ciq257

[B5] Lopardo G Fridman D Gonzalez Arzac M Calmaggi A Smayevsky J Podesta O Clara L Uropathogen resistance: are laboratory-generated data reliable enough? J Chemother 2007 14 33 37 10.1179/joc.2007.19.1.33 17309848

[B6] Stamm WE Counts GW Running KR Fihn S Turck M Holmes KK Diagnosis of coliform infection in acutely dysuric women N Engl J Med 1982 14 463 468 10.1056/NEJM198208193070802 7099208

[B7] Société Française de Microbiologie Rémic. Référentiel en Microbiologie Médicale Volume 1 2012 4 PARIS: Société Française de Microbiologie

[B8] McIsaac WJ Moineddin R Ross S Validation of a Decision Aid to Assist Physicians in Reducing Unnecessary Antibiotic Drug Use for Acute Cystitis Arch Intern Med 2007 14 2201 2206 10.1001/archinte.167.20.2201 17998492

[B9] Hummers-Pradier E Ohse AM Koch M Heizmann WR Kochen MM Management of urinary tract infections in female general practice patients Fam Pract 2005 14 1 71 77 15640290 10.1093/fampra/cmh720

[B10] McIsaac WJ Low DE Biringer A Pimlott N Evans M Glazier R The impact of empirical management of acute cystitis on unnecessary antibiotic use Arch Intern Med 2002 14 600 605 10.1001/archinte.162.5.600 11871930

[B11] Christiaens TCM De Meyere M Verschraegen G Peersman W Heytens S De Maeseneer JM Randomised controlled trial of nitrofurantoin versus placebo in the treatment of uncomplicated urinary tract infection in adult women Br J Gen Pract 2002 14 729 734 12236276 PMC1314413

[B12] Bent S Nallamothu BK Simel DL Fihn SD Saint S Does this woman have an acute uncomplicated urinary tract infection? JAMA: The Journal of the American Medical Association 2002 14 2701 2710 10.1001/jama.287.20.2701 12020306

[B13] Giesen LGM Cousins G Dimitrov BD van de Laar FA Fahey T Predicting acute uncomplicated urinary tract infection in women: a systematic review of the diagnostic accuracy of symptoms and signs BMC Fam Pract 2010 14 78 10.1186/1471-2296-11-78 20969801 PMC2987910

[B14] Foxman B Barlow R D’Arcy H Gillespie B Sobel JD Urinary tract infection: self-reported incidence and associated costs Ann Epidemiol 2000 14 509 515 10.1016/S1047-2797(00)00072-7 11118930

[B15] Little P Turner S Rumsby K Jones R Warner G Moore M Lowes JA Smith H Hawke C Leydon G Mullee M Validating the prediction of lower urinary tract infection in primary care: sensitivity and specificity of urinary dipsticks and clinical scores in women Br J Gen Pract 2010 14 495 500 10.3399/bjgp10X514747 20594439 PMC2894378

[B16] Pitout JD Laupland KB Extended-spectrum β-lactamase-producing Enterobacteriaceae: an emerging public-health concern Lancet Infect Dis 2008 14 159 166 10.1016/S1473-3099(08)70041-0 18291338

[B17] Hansen DS Schumacher H Hansen F Stegger M Hertz FB Schønning K Justesen US Frimodt-Møller N Extended-spectrum β-lactamase (ESBL) in Danish clinical isolates of Escherichia coli and Klebsiella pneumoniae: Prevalence, β-lactamase distribution, phylogroups, and co-resistance Scand J Infect Dis 2012 14 174 181 10.3109/00365548.2011.632642 22364227

[B18] Ben-Ami R Rodríguez-Baño J Arslan H Pitout JDD Quentin C Calbo ES Azap OK Arpin C Pascual A Livermore DM Garau J Carmeli Y A multinational survey of risk factors for infection with extended-spectrum beta-lactamase-producing enterobacteriaceae in nonhospitalized patients Clin Infect Dis 2009 14 682 690 10.1086/604713 19622043

[B19] Rodríguez-Baño J Picón E Gijón P Hernández JR Ruíz M Peña C Almela M Almirante B Grill F Colomina J Giménez M Oliver A Horcajada JP Navarro G Coloma A Pascual A Community-Onset Bacteremia Due to Extended-Spectrum β-Lactamase–Producing Escherichia coli:Risk Factors and Prognosis Clin Infect Dis 2010 14 40 48 10.1086/649537 19995215

[B20] Briongos-Figuero LS Gómez-Traveso T Bachiller-Luque P Domínguez-Gil González M Gómez-Nieto A Palacios-Martín T González-Sagrado M Dueñas-Laita A Pérez-Castrillón JL Epidemiology, risk factors and comorbidity for urinary tract infections caused by extended-spectrum beta-lactamase (ESBL)-producing enterobacteria Int J Clin Pract 2012 14 891 896 10.1111/j.1742-1241.2012.02991.x 22897466

[B21] Aubin C Does This Woman Have an Acute Uncomplicated Urinary Tract Infection? Ann Emerg Med 2007 14 106 108 10.1016/j.annemergmed.2006.09.022 17203544

[B22] Hooton TM Clinical practice. Uncomplicated urinary tract infection N Engl J Med 2012 14 1028 1037 10.1056/NEJMcp1104429 22417256

[B23] Gupta K Hooton TM Roberts PL Stamm WE Short-course nitrofurantoin for the treatment of acute uncomplicated cystitis in women Arch Intern Med 2007 14 2207 2212 10.1001/archinte.167.20.2207 17998493

[B24] Société Française de Microbiologie Société Française de Microbiologie` Recommandations 2011 du Comité de l'antibiogramme de la Société Française de Microbiologie 2012 Paris

[B25] Falagas ME Vouloumanou EK Togias AG Karadima M Kapaskelis AM Rafailidis PI Athanasiou S Fosfomycin versus other antibiotics for the treatment of cystitis: a meta-analysis of randomized controlled trials J Antimicrob Chemother 2010 14 1862 1877 10.1093/jac/dkq237 20587612

[B26] Pitkäjärvi T Pyykönen ML Kannisto K Piippo T Viita P Pivmecillinam treatment in acute cystitis. Three versus seven days study Arzneimittelforschung 1990 14 1156 1158 2291755

[B27] De Backer D Christiaens T Heytens S De Sutter A Stobberingh EE Verschraegen G Evolution of bacterial susceptibility pattern of Escherichia coli in uncomplicated urinary tract infections in a country with high antibiotic consumption: a comparison of two surveys with a 10 year interval J Antimicrob Chemother 2008 14 364 368 10.1093/jac/dkn197 18499768

[B28] Hovelius B Mårdh PA Nygaard-Pedersen L Wathne B Nalidixic acid and pivmecillinam for treatment of acute lower urinary tract infections Scand J Prim Health Care 1985 14 227 232 10.3109/02813438509013954 4081404

[B29] Schito GC Naber KG Botto H Palou J Mazzei T Gualco L Marchese A The ARESC study: an international survey on the antimicrobial resistance of pathogens involved in uncomplicated urinary tract infections Int J Antimicrob Agents 2009 14 407 413 10.1016/j.ijantimicag.2009.04.012 19505803

[B30] Nicolle LE Pivmecillinam for the treatment of acute uncomplicated urinary infection Int J Clin Pract 1999 14 612 617 10692756

[B31] Neuzillet Y Naber KG Schito G Gualco L Botto H French results of the ARESC study: clinical aspects and epidemiology of antimicrobial resistance in female patients with cystitis. Implications for empiric therapy Med Mal Infect 2012 14 66 75 10.1016/j.medmal.2011.07.005 22264668

[B32] Sabuncu E David J Bernède-Bauduin C Pépin S Leroy M Boëlle P-Y Watier L Guillemot D Significant reduction of antibiotic use in the community after a nationwide campaign in France, 2002–2007 PLoS Med 2009 14 e1000084 10.1371/journal.pmed.1000084 19492093 PMC2683932

[B33] Holmberg L Boman G Böttiger LE Eriksson B Spross R Wessling A Adverse reactions to nitrofurantoin. Analysis of 921 reports Am J Med 1980 14 733 738 10.1016/0002-9343(80)90443-X 7435512

[B34] Linhares IS Raposo T Rodrigues AN Almeida A Frequency and antimicrobial resistance patterns of bacteria implicated in community urinary tract infections: a ten-year surveillance study (2000–2009) BMC Infect Dis 2013 14 1 1 10.1186/1471-2334-13-1 23327474 PMC3556060

[B35] Fabre R Mérens A Lefebvre F Epifanoff G Cerutti F Pupin H Tardif D Cavallo JD Ternois I Susceptibility to antibiotics of Escherichia coli isolated from community-acquired urinary tract infections Med Mal Infect 2010 14 555 559 10.1016/j.medmal.2010.03.002 20417046

[B36] Vellinga A Tansey S Hanahoe B Bennett K Murphy AW Cormican M Trimethoprim and ciprofloxacin resistance and prescribing in urinary tract infection associated with Escherichia coli: a multilevel model J Antimicrob Chemother 2012 14 2523 2530 10.1093/jac/dks222 22729920

